# Epidemiological investigation of caries prevalence in first grade school children in Rhineland-Palatinate, Germany

**DOI:** 10.1186/s13005-015-0091-8

**Published:** 2015-10-02

**Authors:** Jens Weusmann, Benjamin Mahmoodi, Adriano Azaripour, Kristian Kordsmeyer, Christian Walter, Brita Willershausen

**Affiliations:** Department of Operative Dentistry, University Medical Center of the, Johannes Gutenberg-University Mainz, Augustusplatz 2, 55131 Mainz, Germany; Department of Oral and Maxillofacial Surgery, Johannes Gutenberg-University, Mainz, Germany

**Keywords:** Caries, Childhood, Epidemiology, Dental health, Prophylaxis

## Abstract

**Introduction:**

The annual examination of first graders’ oral health as stipulated by law aimed to reach every child in Rhineland-Palatinate (Germany) in their first year of school. We intended to evaluate the first graders’ oral health based on the examination data for 2013/2014.

**Methods:**

Instructed examiners measured the d3mft(deciduous)/D3MFT(permanent) index according to World Health Organization criteria in 25,020 predominantly 6–7 year-old first-grade school children. Only caries affecting dentin was diagnosed; no radiography or fiber-transillumination was used. Out of the d3mft value, the “Significant Caries Index” (SiC) was calculated. This index identifies the dmft score of the third of the population with the highest caries experience. Descriptive analysis was performed.

**Results:**

Out of the the examined children, 60.9 % were caries free. Mean d3mft score was 1.28 ± 2.27 while the mean SiC was 3.73 ± 2.51. A distinctly higher d3mft was found in the decidous molars compared to the front teeth. Boys were significantly more caries-experienced than girls (*p* < 0.001).

**Conclusion:**

The results of this study confirm the lasting trend towards decreasing caries prevalence in children starting school found in previous cross-sectional studies. This trend was observed in the high-risk group (obtained by SiC) as well as in the entire study population. Particular attention in caries prophylaxis should be paid to the primary molars.

## Introduction

Dental caries, as a result of a disturbance of the ecological balance on the dental hard tissues caused by plaque microorganisms [1] is one of the most prevalent diseases in children. Among 5- to 17-year-old Americans, it is more than 5 times as common as reported asthma and 7 times as common as hay fever [2]. In 1981, one of the global goals of the World Health Organization and World Dental Federation was to reach a 50 % caries free dentition in five-and-six year-old children until the year 2000. This aim was, in a global view, achieved widely [3].

In 2003, World Health Organization, World Dental Federation and International Association for Dental Research suggested to consider the specific values “with regard of the political, socio-economic and legislative context” and left the decision about the definite aims to the national level [3]. Thus, the German dental board aims 80 % of the six-year old children in Germany to have a caries free primary dentition in 2020 [4].

Having high caries experience early in life predicts having an increased risk of caries in adulthood [5]. Hence, prevention plays an important role already in childhood [6]. As caries can, depending on the extent, negatively affect the children’s ability to eat, sleep, and do schoolwork, therefore, it’s effect on the overall aims of children’s development should not be underestimated [6].

Dental public health promotion programs for kindergartens and schools are legally regulated in Austria and Germany [7]. The German Association for Youth Dental Healthcare coordinates the prevention programs on a national level, while regional branches work together with schools and kindergartens on a local level.

The aim of this study was to examine caries prevalence and experience as well as localisation in first grade school children in the german state of Rhineland-Palatinate.

## Methods

Rhineland-Palatinate is a state in the Federal Republic of Germany with approximately four millions inhabitants. Here, within the scope of prevention measures required by law, every year the first grade school children (six and seven years-old) are systematically examined by dentists from the German association for youth dental healthcare. The d3mft/D3MFT index was evaluated.

The dmft/DMFT index has been in use for about 75 years and still remains the most commonly used epidemiological index for assessing dental caries [8]. The letter d/D means a **d**ecayed tooth, m/M a **m**issing tooth extracted due to caries and f/F stands for a tooth with an intact **f**illing; lowercase letters were used for the primary and capital letters for the permanent dentition. The d3mft is a modification that assesses caries only if the decay affects the dentin layer.

To estimate the burden of the high risk groups populations the Significant Caries Index (SiC) first described by Bratthall is used, describing the the mean dmft of the third of the population with the highest caries experience [9].

The aim of this cross-sectional study was to examine the oral health of all first year elementary school children in the federal state of Rhineland-Palatinate, Germany in 2013/2014 and to analyse the distribution of caries. For this purpose, we aimed to include all available records made by the school dentists in 2013/2014.

A total of 541 school dentists contributed to the findings of this study. The examiners were briefed by a brochure with guidelines concerning caries detection that are based on the WHO criteria. The examinations were conducted between September 2013 and June 2014. Children in 844 primary schools were included in this study.

The examinations took place at school in separate rooms. The children were seated on a normal chair. A disposable mouth mirror was used by default, as well as a standartised lamplight. No radiography or fiber-optic transillumination was used. Dental probing was done if dentin involvement was uncertain only.

Every child was categorised into either “naturally healthy”, which was defined as d3mft + D3MFT = 0; “successfully treated/ no need for treatment” what means F(T)/f(t) > 0 simultaneously to d3m(t)/D3M(T) = 0, or “need for treatment”, if d3(t)/D3(T) > 0. An “increased risk of caries” was diagnosed if d3mft + D3MFT > 5 and/or D3(T) > 0.

The parents had been informed in writing in advance about the screening. Afterwards, they were informed about the results by a letter and, if necessary, suggested to consult a dentist with their child.

Since the examination was not carried out for a primary scientific purpose, no ethical approval was necessary. This was confirmed by a letter from the Rhineland-Palatinate ethics committee, dated 05-13-15. All paper screening forms were collected at the Rhineland-Palatinate Association for Youth Dental Healthcare and were digitalised anonymously using MS Excel.

Mann–Whitney U tests (two-sided) were done to compare dmft and SiC of male and female children; a two-sided chi-square test was conducted to compare the number of male and female children with dmft = 0. IBM SPSS 22.00.00 was used for analysing the data. The global level of significance was set at 5 %; due to multiple testing the Bonferroni correction was applied. The local level of significance was 0.017.

## Results

### Study population

**Fig. 1:**
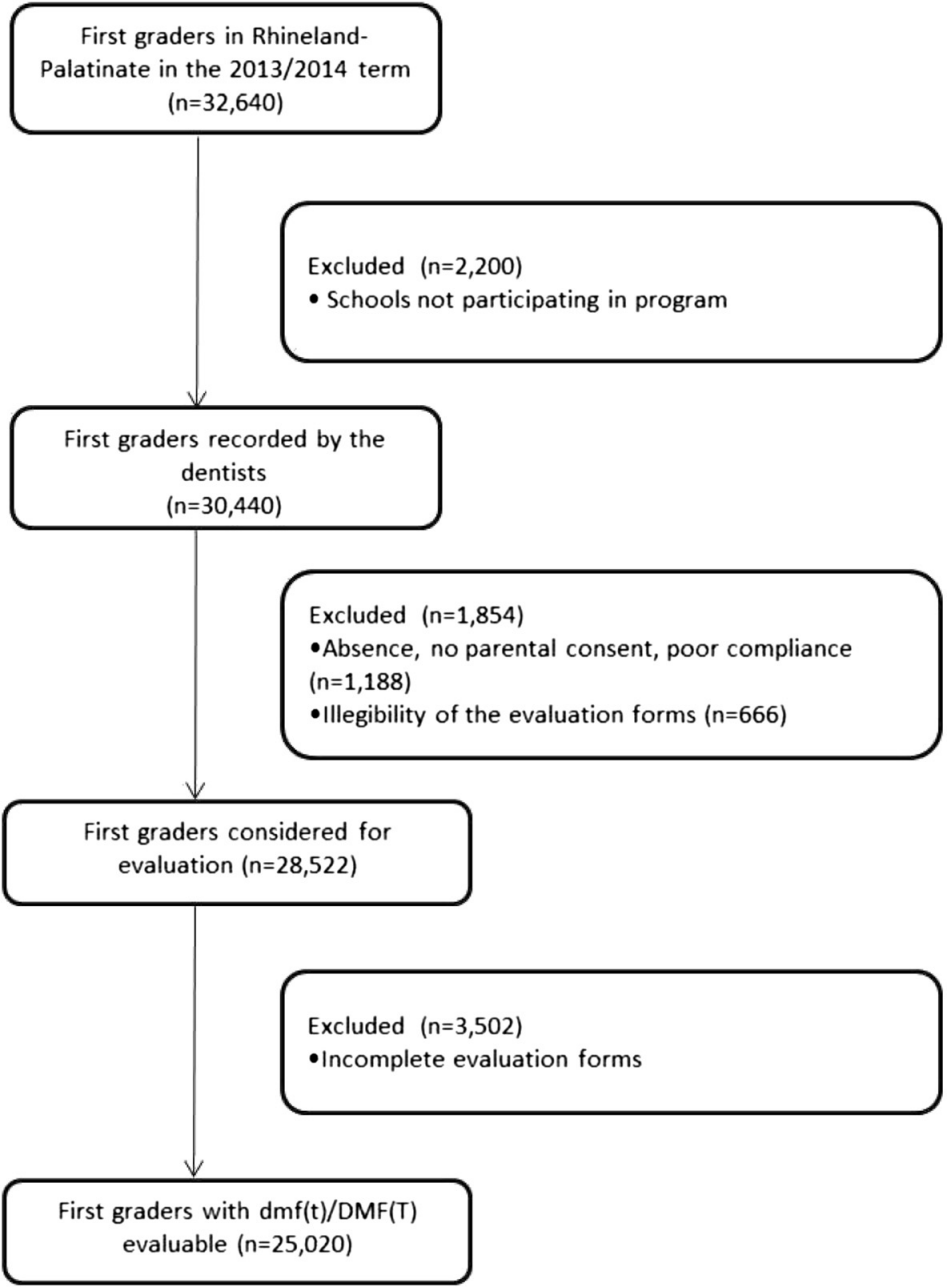
Study participants flow diagram

According to the governmental data, 32,640 first graders were registered in Rhineland-Palatinate for the school term 2013/2014 in a total of 825 elementary schools. Out of those first graders, 30,376 (93 %) were recorded by the school dentists. 1,854 (5.7 %) individuals were excluded due to lack of compliance, absence, no parental consent or illegibility of the examination sheets resulting in 28,522 students from 825 schools. For another 3,502, the detailed d3mft/D3MFT findings were not available, so that for these analyses only 25,020 subjects were included (Fig. 1).

### Oral health evaluation

50.9 % of the children were male. The proportion of female children with naturally healthy teeth was 63.5 % compared to 59.2 % in male children.

Since 4,118 first graders were not recorded due to non-participation or illegibility (Fig. 1), the oral health evaluation was available for 28,522 first graders (Table 1). 25.9 % of the children had caries that needed to be treated. Out of those 7.7 % (equaling 1.9 % from the entire population; boys 8.2 %, girls 6.7 %) had an increased caries risk (d3mft > 5).

**Table 1:** Oral health estimation of first graders in Rhineland-Palatinate in 2013/2014 in percent (n = 28,522)

Dental condition	%	n
Naturally healthy	60.9	17,364
Successfully treated	13.2	3,763
Need for caries treatment	25.9	7,395
Increased Risk of Caries	7.7	2,188

### d3mft, D3MFT and SiC

From a total of n = 25,020 individuals, the original examination sheets were evaluable in detail, including d3mft/D3MFT (Fig. 1). 50.9 % of the children were male, 49.1 % were female.

**Fig. 2:**
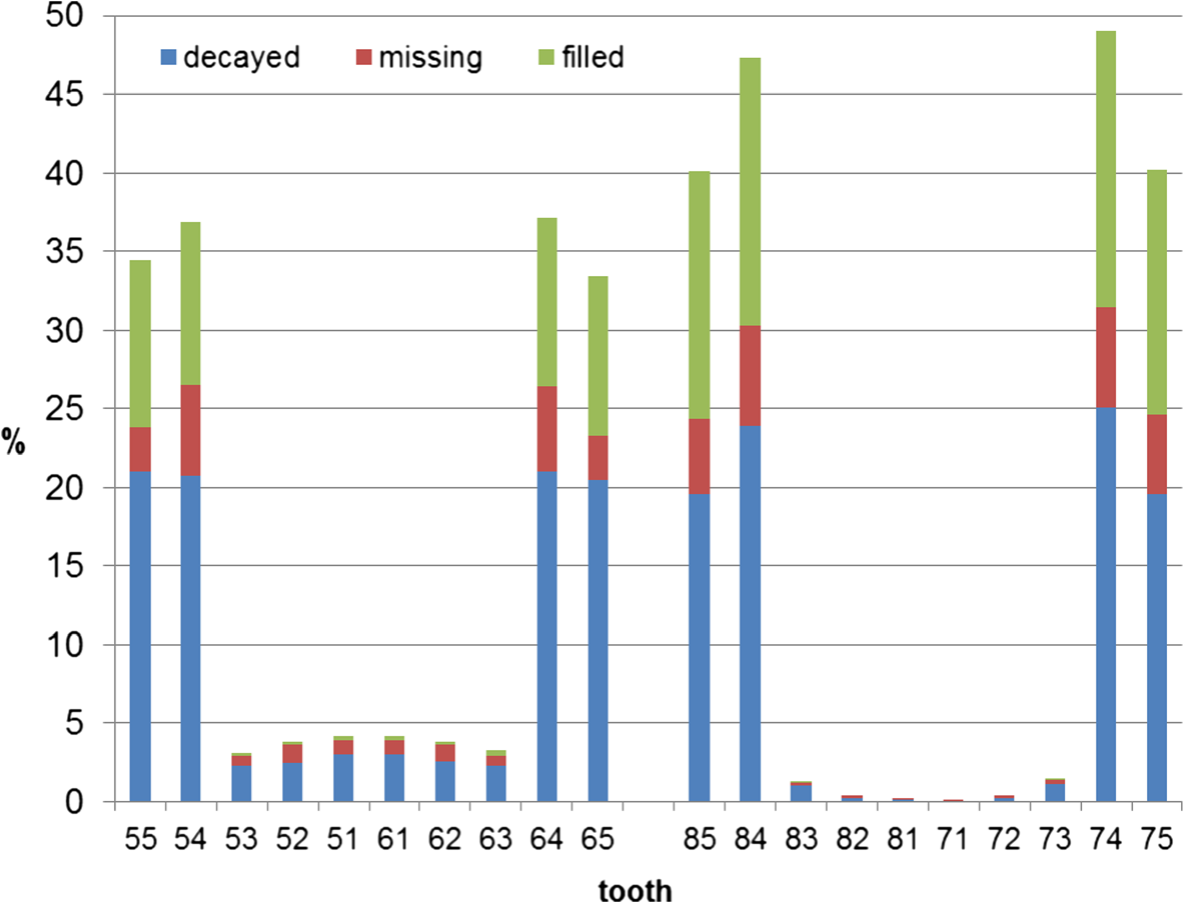
Distribution of the d3mft index (n = 25,020) according to teeth

The mean d3mft/D3MFT results are presented in Table 2. Figure 2 shows the distribution of the d3mft findings with respect to the intra-oral position.

**Table 2:** School children’s d3mft/D3MFT and SiC

Primary dentition	dmf(t)	d(t)	m(t)	f(t)	SiC (dmft)
	1.28 (SE:0.014)	0.71	0.17	0.41	3.73
Permanent dentition	DMF(T)	D(T)	M(T)	F(T)	SiC (DMFT)
	0.04 (SE:0.02)	0.03	0.0007	0.009	0.08

The better overall dental health of girls compared to boys is also seen in the d3mft distribution. The group of girls with a d3mft = 0 was 65.3 % compared to 60.8 % (*p* < 0.001). At the same time, the proportion of boys with a d3mft score of 10 or higher with 1.2 % was twice as high as that of girls. Also the mean d3mft was 1.38 (SD: 2.37, Q1 = 0, median = 0, Q3 = 2) compared to 1.15 (SD: 2.12, Q1 = 0, median = 0, Q3 = 2) was higher among male than among female students (*p* < 0.001), as well as the SiC (3.97 [SD: 2.58, Q1 = 2, median = 3, Q3 = 6] vs. 3.41 [SD: 2.40, Q1 = 2, median = 3, Q3 = 5]) (p = 0.004). The d3mft ranged from 0 to 20 and the D3MFT from 0 to 6 with a continuous decrease the higher the index was. Only minor variations were seen in children with very high d3mft/D3MFT index (>15). In the present study the state of teeth eruption was not evaluated.

Looking at the location of caries in the deciduous dentition, a clear symmetry and an accumulation in the molar area is obvious (Fig. 2).

## Discussion

The average d3mft score among first graders in Rhineland-Palatinate in this study was 1.28 with 62.7 % with a caries free primary dentition (d3mft = 0). This confirms a trend towards lower caries prevalence as seen in the last years. The German Association for Youth Dental Healthcare evaluated the dmft of six-and-seven year-old children in Germany, including Rhineland-Palatinate in the years 1994/1995, 1997, 2000, 2004 and 2009 [7]. In 2009, 55.5 % of the children with a caries free primary dentition [7]. From 1994/1995 until 2009 the mean dmft decreased by 36.4 %.

The d3mft of 1.28 of our study reflects another decline of 28.1 % compared to the recent values of Rhineland-Palatinate [7] similar to the average SiC value that decreased 24.8 % from 4.96 in 2009 to 3.73 in 2014, with the limitation of the differing diagnostic procedure.

Still, no caries assessment method has gained acceptance for international studies. Discrepancies were furthermore to be found in the sample selection compared to the former studies: In the 2009 German Association for Youth Dental Healthcare survey, a 5 % sample (n = 2,096) was selected using a two-stage random sampling procedure [7] contrary to our attempt to include all available data.

The aims for caries prevalence in Germany postulated by the German Dental Board aimed 80 % of the six year-old children to be caries free in 2020 [4]. Although this goal seems still far away, the ongoing trend towards lower caries prevalence in first graders is undeniable.

The d3mft accumulation in the deciduous molars, primarily in the lower jaw is noticeable. Children as well as their parents should call special attention to dental hygiene measures. Causative for that finding could be the motoric deficiencies in an early age, combined with the increased difficulty to keep the posterior interdental spaces clean. Additionally, the lack of visibility compared to the front teeth could be a reason for the neglect. Even though it remains uncertain, whether this is truly a problem of insufficient oral hygiene or caused by other factors such as anatomy, it would be of interest to clearly identify the reason for this finding.

The main limitation of our study is the large number of examiners and the lack of inter-examiner reliability tests. Further limits of the study might be the data acquisition, as well as the slightly differing way of diagnosing caries, since in our study dentin affection was decisive. Usually, the dmft/DMFT score is obtained in a setting without a dental chair with the appropriate light and air syringe. Within the last decades, the use of tooth-coloured restaurations such as compomeres, composites, and modern glass ionomere materials has increased and is part of the dental teaching curricula in many countries [10]. Therefore, tooth-coloured fillings that meet the highest standards of esthetics are more difficult to diagnose in a screening situation and could have influenced the results towards a lower dmft score. Unfortunately, no international standard to determine the decay in dmft has been established yet. The range in other studys reaches from “no exact caries definition” over “cavitating lesions” to “caries affecting the dentin layer”, as conducted in our study. This, as well as the aspect that caries lesions affecting only enamel were not detected in this study, will have lead to a lower dmft score.

Nonetheless, with respect to the high number of examiners, “dentin affection” is comparatively easy to reproduce and could have helped to avoid false positive diagnoses.

A more differentiated caries index, as well as measured interexaminer-reliabilities would clearly have a positive influence on the findings’ validity. Looking at the high number of first graders and the high number of examiners, the latter postulation will be most difficult to establish. Several field studies used ICDAS as an assessment method [11, 12]. This index provides findings that give a statement about caries quality instead of a sheer yes/no-decision.

In the year 2000, Pistorius *et al.* independently analysed the dental health of 3,880 first grade school children out of 80 schools in Rheinhessen, a small region of Rhineland-Palatinate within the scope of governmental group prophylaxis [13]. In this study, dental probes were used for caries detection. The average dmft score of 1.9 with a fraction of 53.4 % having a naturally healthy dentition confirms the overall trend between 1994 (39.2 % caries free) up today (62.7 % caries free).

Looking at the permanent dentition, the 2009 study has, with a DMFT score of 0.03, similar findings to our result of 0.04. The comparatively high DMFT value of 0.19 found in 2000 by Pistorius *et al.* may result of the dental probing that also lead to a caries diagnose in first molars with deep fissures that were not sealed at the time of the examination. Since caries is a process that needs a longer period of time until clinical manifestation, it is hardly surprising that the DMFT was very low. It would have been interesting to know the amount of erupted permanent teeth in this population.

In an international comparison, dmft and caries experience in our findings were at a low level. Table 3 gives an overview of the available results published in comparable studies within the recent years. However, this is to state with the limitation that most of the studies were conducted in developing countries or structurally weak areas.

**Table 3:** Overview on other recent studies with 6-year-old children

Name	Country	Sample size	Mean dmft	Caries experience(%)
Rajab [17]	Jordan	2496	3.3	76.4
Prasaj Dixit [18]	Nepal(Chitwan)	n.a.	1.59	52.0
Hashim [19]	UAE(Ajman)	429	1.12	n.a.
Tadakamadla [20]	India(Udaipur)	875	1.69	58.9
Liu [21]	China(Sechuan)	714	3.94	74.37
Basha [22]	India(Davangere)	196	3.2	50.51
Gorbatova [23]	Russia (Archangelsk)	532	6.71	93.4

Regarding the reasons for the ongoing decrease of the dmft, it is, by nature, difficult to pinpoint the exact causes. The systematic educational work in schools and kindergartens might be a reason for the children’s caries decline. In that context, school and preschool children are taught about brushing methods, professional prophylaxis at the dentist’s office, the use of fluorides and tooth-protective nutrition. Parental habits, knowledge and attitude play a distinct role for the child’s caries prognosis [14]. Possibly, today’s six-year-olds profit from the educational work in their parent’s childhood and adolescence. In Germany, the market share of fluoridated salt rose from 20 % in 1997 to just below 70 % since 2007. As salt is a simple and effective prevention method, this could be another cause for the caries decrease [15].

Even though childhood caries underwent an enormous reduction within the last years, this fact should not be taken for granted, since numerous studies show a connection between children’s socioeconomic status and caries risk [16]. Research on the reasons for the intra-oral caries polarisation would be of interest and might lead to therapeutic consequences in future prophylaxis and treatment of deciduous teeth.

## Conclusions

The trend towards lower caries prevalence in Rhineland-Palatinate continues. Male first graders have significantly more caries experience than their female schoolmates, both in the fraction with low and the one with high caries activity. Prophylaxis efforts should specially focus on the primary molars, since the d3mft values were remarkably higher in this area.
